# Supplementary material for: Expression and clinical value of EGFR in human meningiomas
Source: PeerJ. 2017 Mar 29;5:e3140. doi: 10.7717/peerj.3140 (PMC5374971; doi:10.7717/peerj.3140)
Supplement: Table S1 — Summary of key clinical records and includes tumor grade, localization, resection grade, patient age and gender. [file peerj-05-3140-s002.docx]

**Table S1: Clinical data.**

| **Clinical Feature** | **All Grades** | **Grade I** | **Grade II** | **Grade III** |
| --- | --- | --- | --- | --- |
| Number of cases (%) | n=186  (100 %) | n=130 (69.9 %) | n=55  (29.6 %) | n=1  (0.5 %) |
| Median age at date of operation | 59 | 58 | 61 | 69 |
| Sex |  |  |  |  |
| Male | 48 | 29 | 18 | 1 |
| Female | 138 | 101 | 37 | 0 |
| F:M | 2.9:1 | 3.5:1 | 2.1:1 | 0:1 |
| Simpson grade |  |  |  |  |
| 1 | 44 | 34 | 10 | 0 |
| 2 | 80 | 53 | 27 | 0 |
| 3 | 30 | 18 | 11 | 1 |
| 4 | 32 | 25 | 7 | 0 |
| Tumor localization |  |  |  |  |
| Falx | 24 | 13 | 11 | 0 |
| Convexity | 86 | 52 | 33 | 1 |
| Basal | 47 | 42 | 5 | 0 |
| Posterior fossa and tentorial | 28 | 23 | 5 | 0 |
| Intraventricular | 1 | 0 | 1 | 0 |
