# Supplementary material for: Expression and clinical value of EGFR in human meningiomas
Source: PeerJ. 2017 Mar 29;5:e3140. doi: 10.7717/peerj.3140 (PMC5374971; doi:10.7717/peerj.3140)
Supplement: Table S2 — The number and percentage of tumors across malignancy grades and their subtypes. [file peerj-05-3140-s003.docx]

**Table S2: Grade and subtype distribution.**

| **Type** | **WHO grade** | **Subtype** | **Frequency** | **Percent** |
| --- | --- | --- | --- | --- |
| Benign | I |  |  |  |
|  |  | Meningothelial | 32 | 17.2 |
|  |  | Fibrous | 12 | 6.5 |
|  |  | Transitional | 78 | 41.9 |
|  |  | Psammomatous | 1 | 0.5 |
|  |  | Angiomatous | 2 | 1.1 |
|  |  | Microcystic | 2 | 1.1 |
|  |  | Secretory | 1 | 0.5 |
|  |  | Lymphoplasmacyte-rich | 1 | 0.5 |
|  |  | Metaplastic | 1 | 0.5 |
| Atypical | II |  | 53 | 28.5 |
|  |  | Clear cell | 2 | 1.1 |
| Anaplastic | III |  | 1 | 0.5 |
| Total |  |  | 186 |  |
