# Supplementary material for: Expression and clinical value of EGFR in human meningiomas
Source: PeerJ. 2017 Mar 29;5:e3140. doi: 10.7717/peerj.3140 (PMC5374971; doi:10.7717/peerj.3140)
Supplement: Table S3 — The table shows p-values (2-tailed exact values) from Mann-Whitney U tests when comparing staining index in tumors with certain histological features to tumors lacking these features. Only grade I tumors are included in these tests. [file peerj-05-3140-s004.docx]

**Table S3: Comparison of antibody SI and histological features** **for grade 1 tumors** (p-values, 2-tailed exact values from Mann-Whitney U tests).

|  | EGFR25 (ICD) | EGFR113 (ECD) | Ph-EGFR | EGF | TGFα |
| --- | --- | --- | --- | --- | --- |
| Mitosis 4+  (n=0) | N/A | N/A | N/A | N/A | N/A |
| Brain infiltration present (n=1) | 1.000 | 0.971 | 1.000 | 1.000 | 1.000 |
| Sheeting present  (n=3) | 0.558 | **0.035** | 0.597 | 0.459 | 1.000 |
| Macronucleoli present  (n=3) | 0.558 | 0.850 | 0.597 | 0.979 | 1.000 |
| Hypercellularity absent  (n=105) | 0.296 | 0.480 | 0.561 | **0.010** | 1.000 |
| Small cell change present  (n=5) | 0.272 | 0.235 | 0.376 | 0.188 | 1.000 |
| Necrosis present  (n=14) | 0.683 | 0.837 | 0.933 | 0.722 | 0.368 |
| Psammoma bodies absent  (n=38) | **0.034** | **0.004** | 0.531 | 0.512 | 0.174 |

Values in bold: statistically significant. Ph-EGFR: phosphorylated EGFR. Brain infiltration: n=34.
