# Supplementary material for: Expression and clinical value of EGFR in human meningiomas
Source: PeerJ. 2017 Mar 29;5:e3140. doi: 10.7717/peerj.3140 (PMC5374971; doi:10.7717/peerj.3140)
Supplement: Table S4 — The p-values (2-tailed exact values) from Mann-Whitney U tests when comparing staining index in tumors with certain histological features to tumors lacking these features. Only grade II tumors are included in these tests. [file peerj-05-3140-s005.docx]

**Table S4: Comparison of antibody SI and histological features** **for grade 2 tumors** (p-values, 2-tailed exact values from Mann-Whitney U tests).

|  | EGFR25 (ICD) | EGFR113 (ECD) | Ph-EGFR | EGF | TGFα |
| --- | --- | --- | --- | --- | --- |
| Mitosis 4+  (n=40) | 0.214 | 0.289 | 1.000 | **0.022** | 1.000 |
| Brain infiltration present (n=12) | 0.116 | 0.078 | 0.510 | 0.777 | 1.000 |
| Sheeting present  (n=10) | 1.000 | 0.421 | 0.584 | 0.765 | 1.000 |
| Macronucleoli present  (n=8) | 0.887 | 0.779 | 0.620 | 0.623 | 1.000 |
| Hypercellularity absent  (n=35) | 0.552 | 0.696 | 0.616 | **0.036** | 1.000 |
| Small cell change present  (n=12) | 0.647 | 0.982 | 1.000 | 0.351 | 1.000 |
| Necrosis absent  (n=30) | 0.539 | 0.558 | **0.037** | 0.483 | 1.000 |
| Psammoma bodies absent  (n=23) | 0.060 | **<0.001** | 0.632 | 0.073 | 1.000 |

Values in bold: statistically significant. Ph-EGFR: phosphorylated EGFR. Brain infiltration: n=31.
