# Supplementary material for: Expression and clinical value of EGFR in human meningiomas
Source: PeerJ. 2017 Mar 29;5:e3140. doi: 10.7717/peerj.3140 (PMC5374971; doi:10.7717/peerj.3140)
Supplement: Table S5 — The weighted kappa statistics between observers for each antibody used in the study. [file peerj-05-3140-s006.docx]

**Table S5: Kappa statistics.**

| **Antibody** | EGFR25 | EGFR113 | Ph-EGFR | TGFα | EGF |
| --- | --- | --- | --- | --- | --- |
| **Weighted kappa** | 0.7621 | 0.5585 | 0.7366 | 0.5678 | 0.7506 |

< 0.00 poor, 0.00 – 0.20 slight, 0.21 – 0.40 fair, 0.41 – 0.60 moderate, 0.61 – 0.80 substantial, 0.81 – 1.00 almost perfect (Landis & Koch 1977).
